# A Rare Case of Crossed Non-fused Ectopic Kidney

**DOI:** 10.7759/cureus.31610

**Published:** 2022-11-17

**Authors:** Rafik Jalal M Ragab, Abdulmajeed Faisal A Albalawi, Saif Atallaha S Alatawi, Fatimah Awadh A Alrasheedi

**Affiliations:** 1 Radiology, Maternity and Children Hospital, Tabuk, SAU; 2 Family Medicine, Al-Rawdah Primary Healthcare Center, Hafr al-Batin, SAU

**Keywords:** renal ectopia, kidney anomaly, unfused, nonfused, case report, ectopic kidney

## Abstract

Crossed and non-fused kidneys are an exceedingly rare congenital anomaly in which one or both kidneys cross the midline to the opposite side. At the same time, the ureters are inserted in their normal anatomical locations in the bladder. Although crossed ectopic kidneys are mostly asymptomatic and incidentally discovered during workup for other disorders, they are prone to urological complications such as urinary tract infections (UTIs). Here we present the case of a 12-year-old male with a history of recurrent UTIs and urinary retention who presented to the emergency department with recurrent UTIs and urinary retention and was eventually found to have an ectopic unfused left kidney in the right lumbar region on computed tomography (CT).

## Introduction

Congenital anomalies of the kidney account for approximately 30% of all prenatal malformations [1]. Renal ectopia is a common malformation leading to an abnormal kidney position. Crossed ectopia occurs when one kidney crosses to the opposite side of the midline while the ureter attaches to the bladder on the same side. Crossed ectopia can be fused with the normal kidney or remain unfused, depending on the fusion of the crossed kidney with the orthotopic kidney. Crossed unfused renal ectopia is an uncommon presentation with a reported incidence of 1 in 75000 autopsies [2]. Most cases are asymptomatic and incidentally discovered on autopsy or during workup for unrelated etiologies. However, it may present with recurrent urinary tract infections (UTIs), urolithiasis, vesicoureteral reflux, or hydronephrosis [3]. In this article, we present the case of a 12-year-old male patient diagnosed with crossed non-fused renal ectopia after presenting with recurrent UTIs.

## Case presentation

A-12-year-old male presented to the emergency department (ED) due to suprapubic pain and discomfort. The patient had a significant history of recurrent UTIs and urinary retention, with no priorly diagnosed genitourinary malformations. His observations on admission were unremarkable, and clinical examination showed mild suprapubic tenderness. Ultrasound of the abdomen and pelvis was conducted, which showed the ectopic placement of the left kidney on the right side and a mild to the moderately distended urinary bladder. Due to urinary retention, the child was catheterized and discharged with a follow-up by the pediatric urologist.

**Figure 1:**
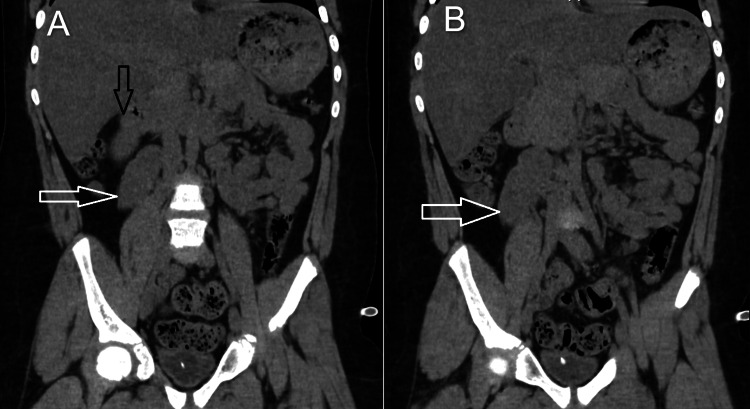
Coronal CT. A) white arrow shows a left ectopic kidney with no gross signs of fusion, and the black arrow is the normal right kidney. B) shows a left ectopic kidney (white arrow)

**Figure 2:**
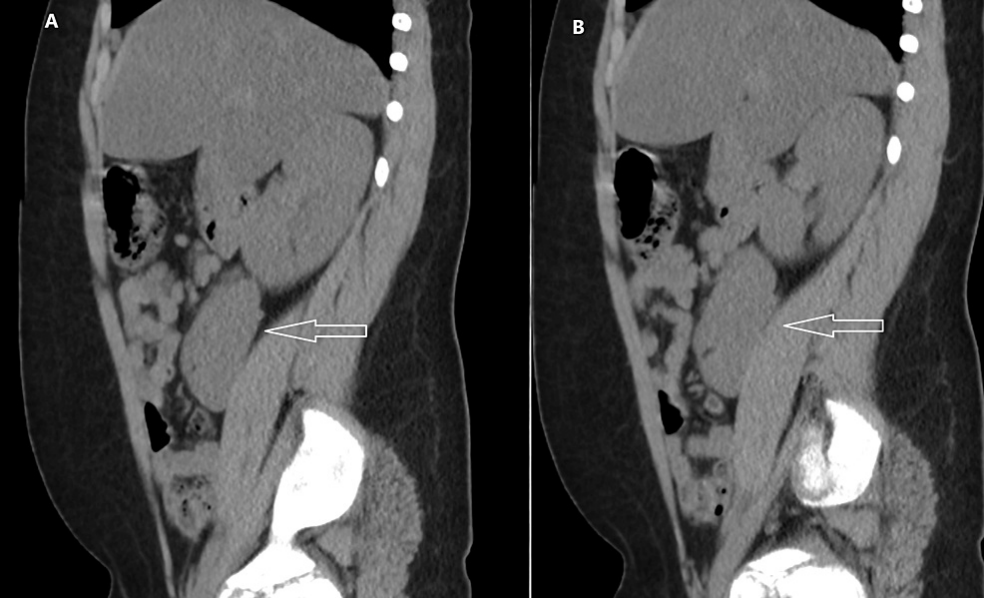
Sagittal CT images (A) and (B) demonstrate crossed renal ectopia (white arrow)

**Figure 3:**
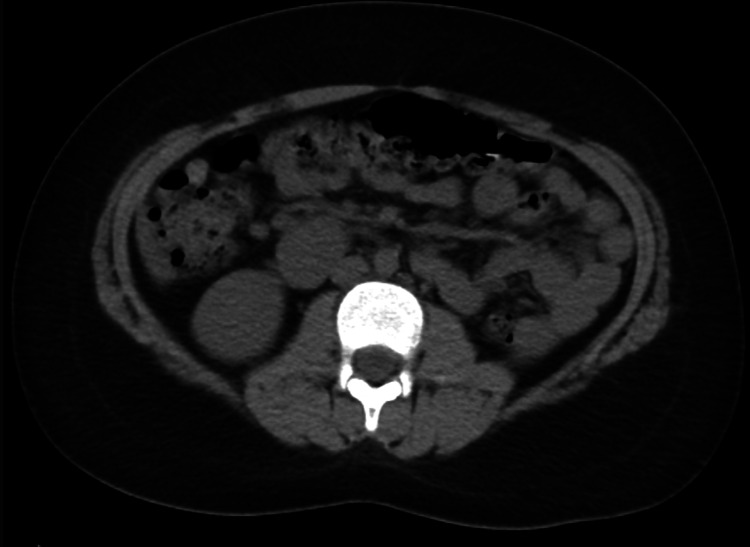
Axial CT image demonstrates crossed left renal ectopia

Three weeks after the initial presentation, the patient presented to the ED again with suspected hematuria. He had no abdominal or flank pain, dysuria, fever, or history of trauma. His vital signs were stable-an unremarkable abdominal examination with no tenderness, rigidity, or guarding. A systemic review of other systems was unremarkable as well. He had been intermittently catheterized following his initial discharge from the ED three weeks ago, and urinalysis revealed that the catheter sample was turbid with no gross hematuria. A CT scan of the abdomen and pelvis was conducted to determine the extent of his renal ectopia and exclude renal stones and other renal pathology (Fig 1-3). The scan showed a left ectopic kidney on the right side of the midline at L3, L4, and L5 lumbar vertebrae levels. The ectopic kidney measured 2.9 x 6.5 x 8 cm at maximum anteroposterior, transverse and craniocaudal diameters, respectively. It was located anterior, inferior, and medial to the right kidney, and there were no gross signs of fusion with the right kidney. The left kidney was located anterior to the right ureter. The left renal pelvis was directed anteriorly and laterally, showing slight fulness; the ureter could not be traced on the left side as this was a non-contrast study. No renal or ureteric stones or hydronephrosis were elicited on imaging, and the right kidney and ureter appeared normal in size and unremarkable. There was no perinephric fat stranding, and the suprarenal glands were normal. A partially filled urinary bladder with a catheter bulb in situ was also noted. The CT scan also showed an enlarged liver with diffuse hypo-attenuation, suggesting possible fatty changes. Multiple enlarged mesenteric lymph nodes, possibly inflammatory, were also noted.

Laboratory investigations showed normal blood urea nitrogen (BUN) levels (3.16 mmol/L) and creatinine levels (36.68 µmol/L), with mild hypokalemia (3.43 mmol/L). In line with the fatty changes in the liver evident on CT, mild transaminitis was also noted with Alanine aminotransferase (ALT) 85.9 U/L and Aspartate aminotransferase (AST) 73.8 U/L (Table 1). Consequent to these findings, a diagnosis was made of crossed-left non-fused renal ectopia, complicated by urinary retention and recurrent UTIs. The patient is currently under management and follow-up by a pediatric urologist.

**Table 1:** Results of laboratory findings

Laboratory test	Results	Normal Range
Alanine aminotransferase (ALT)	85.9 U/L	10-45 U/L
Aspartate aminotransferase (AST)	73.8 U/L	5-46 U/L
Sodium	136 mmol/l	136-145 mmol/l
Potassium	3.43 mmol/l	3.5-5.1 mmol/l
Blood Urea Nitrogen	3.16 mmol/l	1.8-6.4 mmol/l
Serum Creatinine	36.68 umol/l	23-70 umol/l

## Discussion

Crossed renal ectopia (CRE) is a rare anomaly resulting from the aberrant migration of the metanephric blastema and ureteric bud to the opposite side of the midline during weeks four to eight of embryological development [4]. In this type of renal ectopia, the ureters remain uncrossed and normally insert into the bladder. The crossed-over kidney may fuse to the orthotopic kidney, leading to the more common crossed-fused ectopia (type A in the McDonald and McClellan classification), with an incidence of 1:2000 autopsies; or remain unfused, leading to a rare non-fused subtype, of CRE (type B), with an incidence of 1:75000 autopsies [5]. CRE is more common in males, and left-to-right-sided crossed ectopia occurs thrice as frequently as right-to-left-sided, as present in our study [6]. Other variants of CRE include solitary CRE (type C) and non-fused bilateral CRE (type D), which are very infrequently encountered in clinical practice [5].

Several hypotheses, such as mechanical theory, genetic theory, and ureteral theory, explain the mechanism behind the development of renal fusion anomalies. The mechanical theory considers renal ectopia to be abnormal positioning of the umbilical arteries due to altered configuration of the arterial fork. This can cause the kidney to migrate to the contralateral side following the least resistance pathway, eventually leading to crossed ectopia [7]. The genetic theory links mutations in the sonic hedgehog (SHH) gene to the development of CRE, which supports that CRE is observed in identical twins and siblings of the same family [8]. The ureteral theory of ectopia proposes that a wandering ureteral bud diffuses with the contralateral metanephric blastema after crossing the midline, leading to a crossed ectopic kidney [5]. According to this theory, the other blastema, which did not fuse with the ureteral bud, eventually regresses. Abnormal lateral flexion and rotation of the caudal end of the embryo can alter the normal position of the blastema relative to the ureteric bud, which can also contribute to the formation of a crossed ectopia. The ureteral and mechanical theories are the most widely accepted mechanisms [9].

After CRE develops, due to a combination of the mechanisms described above, it is difficult to determine the exact factors which promote fusion or non-fusion. The rare incidence of non-fused CRE suggests that the mechanisms leading to CRE development may also promote fusion in most cases [9].

Non-fused CRE can present clinically in different ways. It may be discovered incidentally during investigations for another disease [5,10]. It can also present with urinary symptoms due to UTIs [11] or an abdominal mass leading to abdominal pain or constipation [3,12]. The crossed kidney may also have renal or ureteric calculi [10]. As with other CREs, non-fused ectopia can also lead to complications such as hydronephrosis, renal volvulus, or recurrent UTIs [13,14]-the last one was the presenting pathology in our patient.

Although non-fused CRE can be identified by ultrasonography, a CT urogram can help to determine the course of the ureter. It can identify any hydronephrosis, hydroureter, renal calculi, or other urinary tract abnormalities [11]. A technetium-diethylenetriamine pentaacetate (99mTc-DTPA) scan can also be used to evaluate kidney function [15]. Laboratory investigations, especially renal function testing, can also be used to determine kidney function in the context of non-fused CRE.

Our case highlights non-fused CRE as the radiological abnormality in a child presenting with recurrent UTIs. It highlights that CT imaging may be required to delineate the extent and fusion of CRE. Pediatric urologists should follow the management of these cases, and kidney function should be closely monitored in these patients.

## Conclusions

Crossed non-fused kidney is a rare congenital renal anomaly in which the kidney is located on the side opposite from which its ureter inserts into the bladder. While ultrasound can indicate renal ectopia, it is necessary to perform CT imaging to differentiate fused or non-fused ectopia, and to identify complications such as renal calculi, pelvic-ureteric junction (PUJ) obstruction, hydronephrosis, and hydroureter, therefore once it's discovered evaluation of the kidney status should be performed. Pediatric urologists should consider non-fused CRE as a potential diagnosis in evaluating recurrent urinary symptoms.
